# Association of GPs’ risk attitudes, level of empathy, and burnout status with PSA testing in primary care

**DOI:** 10.3399/bjgp15X687649

**Published:** 2015-11-06

**Authors:** Anette F Pedersen, Anders H Carlsen, Peter Vedsted

**Affiliations:** Research Unit for General Practice and Research Centre for Cancer Diagnosis in Primary Care (CaP), Aarhus University, Aarhus, Denmark.; Research Unit for General Practice and Research Centre for Cancer Diagnosis in Primary Care (CaP), Aarhus University, Aarhus, Denmark.; Research Unit for General Practice and Research Centre for Cancer Diagnosis in Primary Care (CaP), Aarhus University, Aarhus, Denmark.

**Keywords:** medical errors, patient safety, primary health care, prostatic neoplasms, psychological, quality of health care, stress

## Abstract

**Background:**

Rates of prostate specific antigen (PSA) test ordering vary among GPs.

**Aim:**

To examine whether GPs’ risk attitude, level of empathy, and burnout status are associated with PSA testing.

**Design and setting:**

Register and questionnaire study including 129 solo GPs (active in the Central Denmark Region) and 76 672 of their adult male patients with no history of or current prostate cancer diagnosis.

**Method:**

PSA tests from 2012 were retrieved from a register and classified as incident (that is, the first PSA test within 24 months), repeated normal, or repeated raised tests. This was merged with information on GPs’ risk attitudes, empathy, and burnout status from a 2012 survey.

**Results:**

Patients registered with a GP with a high score on anxiety caused by uncertainty (odds ratio [OR] 1.03, 95% confidence interval [CI] = 1.00 to 1.06, *P* = 0.025) or concern about bad outcomes (OR 1.04; 95% CI = 1.00 to 1.08, *P* = 0.034) were more likely to have an incident PSA test, whereas those registered with a GP with increased tolerance for ambiguity were less likely (OR 0.98, 95% CI = 0.96 to 1.00, *P* = 0.025). Patients registered with a GP reporting high tolerance for ambiguity (OR 0.96, 95% CI = 0.94 to 0.99, *P* = 0.009) or high propensity to risk-taking (OR 0.97, 95% CI = 0.93 to 1.00, *P* = 0.047) were less likely to have a repeated normal PSA test.

**Conclusion:**

Various aspects of GPs’ risk-taking attitudes were associated with patients’ probability of having an incident and a repeated normal PSA test. The probability of having a repeated raised PSA test was not influenced by any of the psychological factors. Burnout and empathy were not associated with PSA testing.

## INTRODUCTION

Rates of prostate specific antigen (PSA) test ordering vary considerably among GPs^1^ and, contrary to international recommendations,^2^ PSA tests are performed on non-symptomatic males, either at a patient’s request or offered by the GP as opportunistic screening for prostate cancer. Only a few of the male patients on a GP’s list develop prostate cancer over a year, but finding them is a challenge for GPs and a fear of overlooking cancer in general seems to be all-pervading.^3^

Risk tolerance has been shown to have an impact on physician activities, for example: longer cardiopulmonary resuscitations in a fictional case,^4^ more use of laboratory services,^5^ and higher admission rate to an intensive care unit.^6^ Because the operationalisation of risk attitude in the cited studies has been criticised for assessing ‘sure-loss-avoidance’ rather than risk tolerance, it seems relevant to examine the effect of risk attitude approached as a multidimensional construct on physician activities.

It has been shown that a GP’s perception of a patient’s need for reassurance is a determinant for test ordering in general practice.^7^ As GPs differ in their capacity to recognise emotions that are being experienced by a patient, it would be relevant to examine whether there is an association between physician empathy and test ordering.

Burnout seems to be increasing among GPs.^8^ The consequences of burnout for patient safety are controversial because burnout has been associated with an increased number of self-reported errors among primary care physicians,^9^ as well as with longer consultations and more focus on problems of a psychosocial nature in the consultations.^10^

In this study it was hypothesised that low levels of risk tolerance are associated with high rates of PSA test ordering, high levels of empathy are associated with high rates of PSA test ordering, and burnout is also associated with rates of PSA test ordering (two-sided hypothesis).

The purpose of the present study was to investigate whether GPs’ PSA test ordering was related to levels of risk attitude, which include tolerance of uncertainty and ambiguity, and also empathy, and burnout.

## METHOD

### Setting

GPs in Denmark are independent contractors with the regional health authorities. The patient list size is on average 1550 patients per GP including children. All Danish citizens are assigned a unique personal identification number, the civil registration number (CRN), from which information from numerous nationwide registers in Denmark can be linked.

### Study population: GPs and patients

In January 2012, all 835 active GPs in the Central Denmark Region (Denmark is organised into five regions and includes several towns; the Central Denmark Region is one of them) were invited to participate in a survey on job satisfaction (‘the GP profile’). GPs were identified by the Regional Registry of Health Providers. Non-responders were sent a reminder after 4 and 13 weeks, and GPs were remunerated to the amount of €50 for responding. For the purpose of this study, only solo GPs (physicians working in single-handed practice) who were active throughout the entire study period (2012) were included.

**How this fits in**

Rates of PSA test ordering vary considerably among GPs. Aspects of GPs’ risk-taking were found to be associated with patients’ probability of having an incident and a repeated normal PSA test, but burnout and empathy were not found to be associated. Uncertainty and ambiguity characterising decision making in general practice may induce discomfort in the GP. GPs might benefit from increased focus on their personal characteristics to reduce stress and empower them to handle such situations.

Study cases were male patients aged ≥20 years who were registered, in the Patient List Register, with one of the included solo GPs throughout 2012. Patients registered with a positive prostate cancer diagnosis before 1 January 2012 were excluded. Information concerning prostate cancer diagnosis (code C619 according to the International Classification of Disease (ICD) version 10) was obtained from The Danish Cancer Register.^11^ Information concerning educational level and marital status for each patient was provided by the Integrated Database for Labour Market Research in Statistics Denmark. According to the International Standard Classification of Education, level of education was classified as low (≤10 years), middle (>10 and ≤15 years), or high (>15 years). Marital status was categorised as cohabiting (married or living with a partner) or single (living alone).

### PSA tests

**Figure 1.**
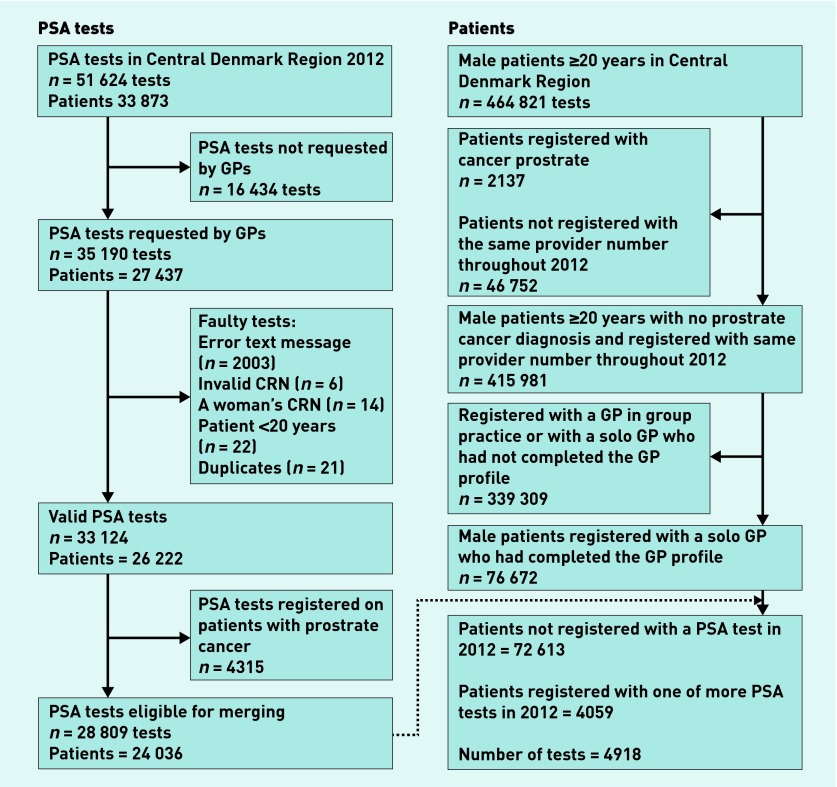
***Flowchart of inclusions of prostate specific antigen (PSA) tests and patients. CRN = civil registration number.***

All PSA tests performed from 1 January to 31 December 2012 in the Central Denmark Region were retrieved from the laboratory database (LABKA).^12^ Data contained patients’ CRN, test results, and identification data of the requesting healthcare provider (see Figure 1 for exclusion of invalid tests). Data were also collected from LABKA concerning PSA tests from 1 January 2010 to 31 December 2011 for the purpose of characterising every PSA test ordered in 2012 as either ‘an incident test,’ that is, the first PSA test within 24 months, ‘a repeated normal test’, that is, a test preceded by a test with a normal result (≤4.0 ng/ml) within the previous 24 months, and ‘a repeated raised test’, that is, a test preceded by a test with a raised result (>4.0 ng/ml) within the previous 24 months.^13^

The pool of PSA tests was merged with study cases by means of patients’ CRN. For every category of the PSA test (incident, repeated normal, and repeated raised), each patient (study case) was registered as having none or one or more of the specific test category performed during 2012.

### GP questionnaire data

Risk attitude was assessed by three scales: the Physician Reaction to Uncertainty (PRU), the Tolerance for Ambiguity (TFA), and the Physician Risk Attitude (PRA). All items were scored on a 6-point Likert scale, and all scales have been developed for use in medical practice.

The PRU scale^14^ consists of four subscales: anxiety due to uncertainty (5 items); concern about bad outcomes (3 items); reluctance to disclose mistakes to physicians (2 items); and reluctance to disclose uncertainty to patients (5 items). Higher scores on each of the subscales reflect more psychological discomfort in dealing with uncertainty. The structure of the scale has been supported by the results of a confirmatory factor analysis.^15^

The TFA scale^16^ includes 7 items, and higher scores indicate more tolerance for ambiguity. The one-factor structure of the scale was revealed by explorative factor analysis.^16^ The PRA scale^17^ consists of 6 items, and higher scores reflect increased risk-seeking behaviour. The items were selected and adapted from the Jackson Personality Index.^18^

Empathy was assessed by the Jefferson Scale of Physician Empathy,^19^ consisting of 20 items scored on a 7-point Likert scale. Higher sum-scores indicate higher levels of empathy. The scale has been validated by explorative factor analysis and test–retest reliability.^19^

Burnout was assessed by the Maslach Burnout Inventory Human-Services-Survey (MBI-HSS).^20^ The MBI-HSS has been used in more than 90% of empirical studies of burnout in the world.^21^ The scale has been translated into Danish according to standardised procedures (forward, backward, expert meetings, and pretest). The MBI-HSS consists of 22 items scored on a 7-point Likert scale constituting three subscales: 1) emotional exhaustion (9 items); 2) depersonalisation (5 items); and 3) personal accomplishment (8 items). Each subscale score is categorised as low or high based on normative population score.^20^ A high level of emotional exhaustion is defined as a score >26, and a high level of depersonalisation is defined as a score >9. Low personal accomplishment is defined as a score <34. Burnout was defined as a high score on the emotional exhaustion subscale and/or a high score on the depersonalisation subscale.^20^

### Analysis

Data were treated as panel data and analysed using mixed-effects logistic regression. Associations between patients’ likelihood of being registered with an incident PSA test, one or more repeated normal PSA tests, or one or more repeated raised PSA tests and GPs’ levels of risk attitude, including tolerance of uncertainty and ambiguity, empathy, and burnout, were calculated as odds ratios (ORs). Each independent variable was included in separate regression models including age, educational level, and marital status of the patients as covariates. As some of the patients were registered with the same GP, the analyses were corrected for clusters using robust variance estimation.

To assist interpretation of results, predictive margins for significant predictor variables were calculated.^22^ The 95% confidence intervals (95% CI) for ratios were calculated and *P*-values ≤5% were considered statistically significant. Data were analysed using STATA (version 13).

## RESULTS

A total of 601 GPs (response rate = 72%) completed and returned the questionnaire. Of these, 129 were included as they were solo GPs and active throughout the entire study period. A total of 76 672 patients met the inclusion criteria and were included as study cases (Figure 1).

The characteristics of the patients are shown in Table 1 and GPs’ scores on the psychological variables and their use of PSA tests are shown in Table 2. Table 3 shows that patient age, educational level, and marital status were strongly associated with having a PSA test performed. Thus, the likelihood of having a PSA test performed rose with increasing age and educational level. Single men were less likely to have a PSA test performed compared with those who were married. These results applied to all three categories of PSA tests.

**Table 1.** Characteristics of the study population, which consisted of males aged ≥20 years registered throughout 2012 with a solo GP who participated in the GP profile study (*n* = 76 672)

**Characteristics of the study population**	

Mean age, years (SD); range	48.9 (17.1); 20–103
Median age (IQI)	48 (35–62)

	***n* (%)**

**Educational level**	
≤10 years	20 554 (26.8)
>10 years and ≤ 15 years	40 153 (52.4)
>15 years	12 742 (16.6)
Missing information	3223 (4.2)

**Marital status**	
Married or cohabiting	50 672 (66.1)
Single	25 391 (33.1)
Missing information	609 (0.8)

**PSA test in 2012**	
Registered with an incident test	2562 (3.3)
Registered with ≥1 repeated normal tests	1154 (1.5)
Registered with ≥1 repeated raised tests	657 (0.9)

IQI = interquartile interval. PSA = prostate specific antigen.

**Table 2.** Characteristics of the solo GPs (*n* = 129)

**Variables**	***n***	**Mean ± SD**	**Observed range**

Age, years	129	55.0 ± 7.0	37–70
Anxiety caused by uncertainty (5 items)	126	13.8 ± 4.2	6–30
Concern about bad outcomes (3 items)	127	7.8 ± 2.9	3–18
Reluctance to disclose mistakes to physicians (2 items)	127	4.1 ± 2.0	2–11
Reluctance to disclose uncertainty to patients (5 items)	126	15.6 ± 4.6	5–30
Tolerance for Ambiguity (7 items)	124	25.7 ± 5.7	11–42
Physician Risk Attitude (6 items)	123	15.7 ± 5.5	6–28
Jefferson Scale of Physician Empathy (20 items)	125	117.2 ± 12.7	77–138

**PSA tests**	***n***	**Median (IQI)**	**Observed range**

Incident	129	17 (11–26)	0–71
Repeated raised	129	6 (3–12)	0–53
Repeated normal	129	6 (4–12)	0–138

The number of female GPs was 32 (24.8%) and the number of male GPs was 96 (74.4%). Information concerning sex was missing for one GP. IQI = interquartile interval. PSA = prostate specific antigen.

**Table 3.** Summary of mixed-effects logistic regression adjusted for patient’s age, level of education, and marital status (*n* = 123 to 129)

	**Incident PSA test**			**≥1 repeated normal PSA tests**			≥**1 repeated raised PSA tests**		

	**OR**	**95% CI**	***P*-value**	**OR**	**95% CI**	***P*-value**	**OR**	**95% CI**	***P*-value**
Patient age, years	1.05	1.05 to 1.06	<0.001	1.07	1.07 to 1.08	<0.001	1.10	1.09 to 1.10	<0.001
		
**Education**									
Low	Ref.			Ref.			Ref.		
Middle	1.25	1.10 to 1.41	<0.001	1.42	1.24 to 1.63	<0.001	1.33	1.09 to 1.61	0.005
High	1.19	1.04 to 1.36	0.011	1.63	1.33 to 1.99	<0.001	1.81	1.42 to 2.29	<0.001
		
**Marital status**									
Cohabiting	Ref.			Ref.			Ref.		
Single	0.78	0.70 to 0.86	<0.001	0.59	0.50 to 0.69	<0.001	0.69	0.56 to 0.85	<0.001
		
**Risk attitudes of GPs**									
Anxiety caused by uncertainty	1.03	1.00 to 1.06	0.025	1.03	0.99 to 1.07	0.128	0.99	0.95 to 1.03	0.631
Concern about bad outcomes	1.04	1.00 to 1.08	0.034	1.03	0.98 to 1.08	0.278	0.98	0.93 to 1.03	0.470
Reluctance to disclose mistakes to physicians	1.01	0.97 to 1.06	0.539	1.03	0.96 to 1.11	0.371	0.98	0.90 to 1.06	0.584
Reluctance to disclose uncertainty to patients	0.99	0.97 to 1.02	0.631	0.96	0.92 to 1.00	0.050	0.98	0.95 to 1.02	0.341
Tolerance for ambiguity	0.98	0.96 to 1.00	0.025	0.96	0.94 to 0.99	0.009	1.00	0.96 to 1.03	0.825
Physician risk attitude	0.99	0.97 to 1.01	0.416	0.97	0.93 to 1.00	0.047	0.99	0.96 to 1.02	0.432
Empathy	1.00	0.99 to 1.01	0.982	1.00	0.99 to 1.01	0.604	1.01	1.00 to 1.02	0.154
		

**Burnout**									
No burnout	Ref.			Ref.			Ref.		
Burnout	1.15	0.90 to 1.48	0.270	1.18	0.85 to 1.63	0.337	1.30	0.96 to 1.77	0.093

OR = odds ratio. PSA = prostate specific antigen. Ref. = reference.

As shown in Table 1, 2562 males were registered with an incident PSA test in 2012 and 430 (16.8%) of these were >4.0 ng/ml.

### Probability of having an incident PSA test

Table 3 shows that patients registered with a GP with a high score on anxiety caused by uncertainty were more likely to have an incident PSA test (adjusted OR 1.03, 95% CI = 1.00 to 1.06, *P* = 0.025). The predicted probability of having an incident PSA test increased from 2.5% to 3.6% (factor 1.44) when comparing GPs in the lowest and highest quartiles of anxiety caused by uncertainty (Table 4). Patients registered with a GP with a high score on concern about bad outcomes were more likely to have an incident PSA test (adjusted OR 1.04; 95% CI = 1.00 to 1.08, *P* = 0.034). Patients registered with a GP with increased tolerance for ambiguity were less likely to have an incident PSA test (adjusted OR 0.98, 95% CI = 0.96 to 1.00, *P* = 0.025).

**Table 4.** Predicted probabilities that a male patient aged ≥20 years will have an incident or a repeated normal PSA test when exposed to a GP scoring in the first, second, third, or fourth quartiles of significant predictor variables

	**First quartile**	**Second quartile**	**Third quartile**	**Fourth quartile**
**Predicted probability of incident PSA test, % (95% CI)**						
Anxiety caused by uncertainty	2.5 (2.0 to 3.0)	2.5 (2.0 to 2.9)	3.0 (2.2 to 3.8)	3.6 (2.8 to 4.4)
Concern about bad outcomes	2.4 (2.0 to 2.9)	3.0 (2.1 to 3.8)	3.1 (2.5 to 3.8)	3.2 (2.4 to 3.9)
Tolerance for ambiguity	3.1 (2.4 to 3.8)	3.7 (2.8 to 4.6)	2.4 (2.0 to 2.8)	2.1 (1.6 to 2.6)

**Predicted probability of repeated normal PSA test, % (95% CI)**						
Tolerance for ambiguity	1.4 (0.9 to 1.9)	1.2 (0.7 to 1.6)	1.0 (0.7 to 1.2)	0.7 (0.5 to 1.0)
Physician risk attitude	1.3 (0.9 to 1.7)	1.2 (0.9 to 1.5)	0.8 (0.5 to 1.1)	0.8 (0.5 to 1.2)

### Probability of having one or more repeated normal PSA tests

Patients registered with a GP reporting increased risk-taking propensity were less likely to have a repeated normal test (adjusted OR 0.97, 95% CI = 0.93 to 1.00, *P* = 0.047) (Table 3). Patients registered with a GP reporting high tolerance for ambiguity were less likely to have a repeated normal test (adjusted OR 0.96, 95% CI = 0.94 to 0.99, *P* = 0.009).

The predicted probability of having one or more repeated normal PSA tests decreased from 1.4% to 0.7% (factor 0.50) when comparing GPs reporting least tolerance for ambiguity with GPs reporting most tolerance for ambiguity (Table 4).

### Probability of having one or more repeated raised PSA tests

Patient likelihood of having one or more repeated raised PSA tests was not associated with any of the psychological factors. GP burnout and empathy were not associated with patient likelihood of having an incident PSA test, a repeated normal test, or a repeated raised test.

## DISCUSSION

### Summary

This study found that adult male patients who were registered with a GP with a high score on anxiety caused by uncertainty and on concern about bad outcomes were more likely to have an incident PSA test during a 1-year follow-up. However, males registered with a GP with high tolerance for ambiguity were less likely to have an incident PSA test. Patients registered with a GP reporting high tolerance for ambiguity also were less likely to have a repeated normal PSA test, as were patients registered with a GP reporting propensity to risk-taking. As recommendations exist concerning the handling of elevated test results, it was found to be reassuring that risk attitudes were not affecting the likelihood of repeated raised PSA tests.

### Strengths and limitations

Among the strengths of the present prospective study are the use of a clinical database for registration of PSA test rates and the classification of PSA into incident tests, repeated normal tests, and repeated raised tests.^13^ Furthermore, from the Danish CRN, it was possible to exclude male patients who had been diagnosed with prostate cancer and are monitored with regular PSA tests. A few methodological weaknesses also have to be mentioned. First, as a consequence of registration of the identity of test orderers in LABKA, only solo GPs could be included in the study. As solo GPs may be a selected group of GPs, biased results in either direction cannot be excluded. Likewise, assessment of risk attitudes, including tolerance of uncertainty and ambiguity, empathy, and burnout, may be influenced by response bias. Second, in the algorithm, a ‘normal PSA test result’ was classified as ≤4.0 ng/ml even though it has been recommended to use age-dependent limits. This may have influenced the categorisation of repeated normal and repeated raised tests, but not the categorisation of incident tests. Third, the scales used for assessment of risk attitude and empathy have all been developed for use in medical practice, but not specifically for use in general practice, and some of the clinical situations used for validation of the scales are far from clinical situations seen in general practice. Fourth, even though the results were adjusted for several patient characteristics, residual confounding cannot be excluded, for example, the influence of GP characteristics that were not considered, such as knowledge and adherence to guidelines.

### Comparison with existing literature

The finding that GPs’ increased risk-taking propensity was one of the factors that reduced patients’ risk of having a repeated normal PSA test accords with results from two previous studies using the same risk-seeking assessment questionnaire. Thus, increased risk-taking propensity was found to be related to lower levels of patient charges used as a proxy for physicians’ resource use,^23^ and to lower admitting rates of emergency room physicians.^17^

The results of one other study revealed an association between higher scores on intolerance of clinical uncertainty and higher scores on reliance on high-technology medicine in medical students and a preference for ‘medicine by numbers.’^24^ Applying this explanation to the present findings, GPs who were intolerant of ambiguity may perceive the PSA test as a way to quantify the risk of prostate cancer in worried patients.

It was found in the present study that patients registered with a GP concerned about bad outcomes were more likely to have an incident PSA test performed. The results of a study including only 20 physicians and using the same scale revealed a non-significant tendency that GPs who were highly concerned about bad outcomes had higher median patient charges than primary care physicians who were less concerned about bad outcomes.^23^

No association was found between burnout and PSA testing. Even though burnout is believed to be a serious issue for the medical profession, evidence concerning the negative influences of burnout on patient safety is actually scarce. The influence of burnout on job performance may depend on the timing of the assessment. Thus, high ambition and perfectionism are risk factors for burnout, and, in the early stages of burnout, a GP’s work may therefore be characterised by thoroughness. In later stages, carelessness and lack of concern for patients’ wellbeing may become dominant. Contingent on the share of early- and late-stage burnt-out GPs in the study, the influence of the one group may counterbalance the influence of the other.

### Implications for practice

Although the absolute effect sizes may seem small, this study documented statistically significant associations between GPs’ risk attitudes and patients’ risk of having an incident PSA test and a repeated normal PSA test. It is important to emphasise that the risk attitudes were scored as continuous factors, and, for instance, an OR of 1.03 means that, for every 1-unit increase in the score, a 3% increase in the odds of taking a PSA test is expected. Therefore, a relatively large difference was seen between the GPs in the first and fourth quartiles. The mean number of incident PSA tests per year taken by the GPs included in this study was 21. As an example, the reported difference in tolerance for ambiguity corresponds to a difference in mean number of incident PSA tests of 11 per year between GPs scoring in the lowest and highest quartiles of the tolerance for ambiguity scale. On a population basis these differences between GPs’ risk-taking may have a large impact on the use of PSA testing in general practice. Furthermore, males in the 25% of practices in which the GPs reported most tolerance for ambiguity had half the probability of getting a repeated normal PSA test compared with males in the 25% of practices in which the GPs had the least tolerance for ambiguity.

Patients often expect to be treated equally and assume that their treatment is based on accepted guidelines. To empower patients to take responsibility for their own health and develop a partnership with their GP, patients should be informed that a GP’s willingness to offer treatment in many instances depends on that GP’s professional opinion, which is influenced by a variety of factors. Medical decision making characterised by uncertainty and ambiguity may induce discomfort in the GP, and GPs might benefit from an increased focus on the strengths and weaknesses of their personality to reduce stress and improve the professional handling of such situations.

## References

[1] Hjertholm P, Fenger-Gron M, Vestergaard M (2015). Variation in general practice prostate-specific antigen testing and prostate cancer outcomes: an ecological study. Int J Cancer.

[2] Cuzick J, Thorat MA, Andriole G (2014). Prevention and early detection of prostate cancer. Lancet Oncol.

[3] Johansen ML, Holtedahl KA, Rudebeck CE (2012). How does the thought of cancer arise in a general practice consultation? Interviews with GPs. Scand J Prim Health Care.

[4] Nightingale SD, Grant M (1988). Risk preference and decision making in critical care situations. Chest.

[5] Nightingale SD (1987). Risk preference and laboratory use. Med Decis Making.

[6] Nightingale SD (1988). Risk preference and admitting rates of emergency room physicians. Med Care.

[7] van der Weijden T, van Bokhoven MA, Dinant GJ (2002). Understanding laboratory testing in diagnostic uncertainty: a qualitative study in general practice. Br J Gen Pract.

[8] Arigoni F, Bovier PA, Sappino AP (2010). Trend of burnout among Swiss doctors. Swiss Med Wkly.

[9] Williams ES, Manwell LB, Konrad TR, Linzer M (2007). The relationship of organizational culture, stress, satisfaction, and burnout with physician-reported error and suboptimal patient care: results from the MEMO study. Health Care Manage Rev.

[10] Zantinge EM, Verhaak PF, de Bakker DH (2009). Does burnout among doctors affect their involvement in patients’ mental health problems? A study of videotaped consultations. BMC Fam Pract.

[11] Gjerstorff ML (2011). The Danish cancer registry. Scand J Public Health.

[12] Grann AF, Erichsen R, Nielsen AG (2011). Existing data sources for clinical epidemiology: the clinical laboratory information system (LABKA) research database at Aarhus University, Denmark. Clin Epidemiol.

[13] Mukai TO, Bro F, Olesen F, Vedsted P (2013). To test or not: a registry-based observational study of an online decision support for prostate-specific antigen tests. Int J Med Inform.

[14] Gerrity MS, DeVellis RF, Earp JA (1990). Physicians’ reactions to uncertainty in patient care. A new measure and new insights. Med Care.

[15] Gerrity MS, White KP, Devellis RF, Dittus RS (1995). Physicians’ reactions to uncertainty: refining the constructs and scales. Motivation Emotion.

[16] Geller G, Tambor ES, Chase GA, Holtzman NA (1993). Measuring physicians’ tolerance for ambiguity and its relationship to their reported practices regarding genetic testing. Med Care.

[17] Pearson SD, Goldman L, Orav EJ (1995). Triage decisions for emergency department patients with chest pain: do physicians’ risk attitudes make the difference?. J Gen Intern Med.

[18] Jackson DN (1975). Jackson personality inventory manual.

[19] Hojat M, Gonnella JS, Nasca TJ (2002). Physician empathy: definition, components, measurement, and relationship to gender and specialty. Am J Psychiatry.

[20] Maslach C, Jackson SE, Leiter MP (1996). Maslach burnout inventory manual.

[21] Kristensen TS, Borritz M, Villadsen E, Christensen KB (2005). The Copenhagen burnout inventory: a new tool for the assessment of burnout. Work Stress.

[22] Williams R (2012). Using the margins command to estimate and interpret adjusted predictions and marginal effects. Stata J.

[23] Allison JJ, Kiefe CI, Cook EF (1998). The association of physician attitudes about uncertainty and risk taking with resource use in a medicare HMO. Med Decis Making.

[24] Merrill JM, Lorimor RJ, Thornby JI, Vallbona C (1998). Reliance on high technology among senior medical students. Am J Med Sci.

